# Comprehensive Quality Control of the Regenerative Therapy Using Platelet Concentrates: The Current Situation and Prospects in Japan

**DOI:** 10.1155/2018/6389157

**Published:** 2018-05-21

**Authors:** Tomoyuki Kawase, Kazuhiro Okuda

**Affiliations:** ^1^Division of Oral Bioengineering, Institute of Medicine and Dentistry, Niigata University, Niigata 951-8514, Japan; ^2^Division of Periodontology, Institute of Medicine and Dentistry, Niigata University, Niigata 951-8514, Japan

## Abstract

Platelet concentrates (PCs), represented by platelet-rich plasma (PRP), have been widely applied in the fields of regenerative and aesthetic therapies. PCs' mechanisms of action, however, are too complicated, and it is not easy to present the whole picture; besides, clinical outcomes are hardly reproducible in many cases. Therefore, several medically advanced countries seemingly intend to regulate PC therapies weakly or strictly because of the increasing popularity. Japan established laws and regulations for PC therapy in the “Act on the Safety of Regenerative Medicine” along with the “Pharmaceuticals, Medical Devices and Other Therapeutic Products Act” in 2014, which, to our knowledge, represent the strictest regulatory framework for production and therapeutic use of PCs in the world. According to these laws and regulations, PCs produced for topical use should be prepared as cell-based medicinal products, essentially as should stem cells, in accordance with their registered (“licensed” under actual conditions) standard operating procedures. Nonetheless, criteria for their quality are not standardized. In this review, we discuss the quality of PC preparations by focusing on the basic concept and regulatory framework of regenerative medicine in Japan. Within the new framework, PC therapy is regulated by a specific notification and registration system, as is stem cell therapy. In comparison with the latter, however, risk factors that hamper successful PC therapy are much fewer. Via appropriate evaluation of patients' conditions and whole-blood samples by simple and sensitive but not yet fully standardized assays, it is theoretically possible that PC quality will be controlled nearly completely. In addition to or instead of standardization of preparation protocols, standardization of preoperative examination of individual PC preparations is an urgent task for improving and guaranteeing the safety and efficacy of PC therapy.

## 1. Introduction

Regenerative therapy using platelet concentrates (PCs), such as platelet-rich plasma (PRP), is an adjuvant biological therapy [1, 2]. Despite more than 2 decades of use of PCs in various areas of regenerative medicine, the essence of PC therapy seems not to be regarded correctly by many clinicians: basically, the mechanisms of its action are not yet fully clarified and wide variations of its clinical outcomes remain to be clearly explained. Partially for these reasons, some regulatory authorities in individual countries are seemingly intending to regulate medical use of PCs by establishing a new regulatory framework. In this review, we introduce the basic concept of the regulatory frameworks for cell-based medicinal products (CBMPs) in Japan, analyze the current situation and understanding of PC therapy, and finally propose the Must-Do list for improving the quality of PC therapy.

## 2. The World Is Changing

### 2.1. Changes in the United States and Europe

The Food and Drug Administration (FDA) of the United States has not yet attempted to regulate but raised some concerns recently over activated PRP [3]. In Europe, the regulatory landscape (related to the products derived from the manipulation of whole blood), which sets forth quality and safety rules for collecting, controlling, processing, preserving, and distributing human blood and its components, is currently governed by Directive 2002/98/EC of the European Parliament and Council of January 27, 2003, and is acknowledged in the various States of the Union with internal regulations [4]. In 2013, the Spanish Agency of Medicines and Medical Devices drew up a comprehensive report and resolution that for the first time regulates the use of PRP as a medicinal product for humans to adapt the applications of PRP to the new requirements of safety and efficacy [5, 6].

### 2.2. Changes in Japan

**Figure 1:**
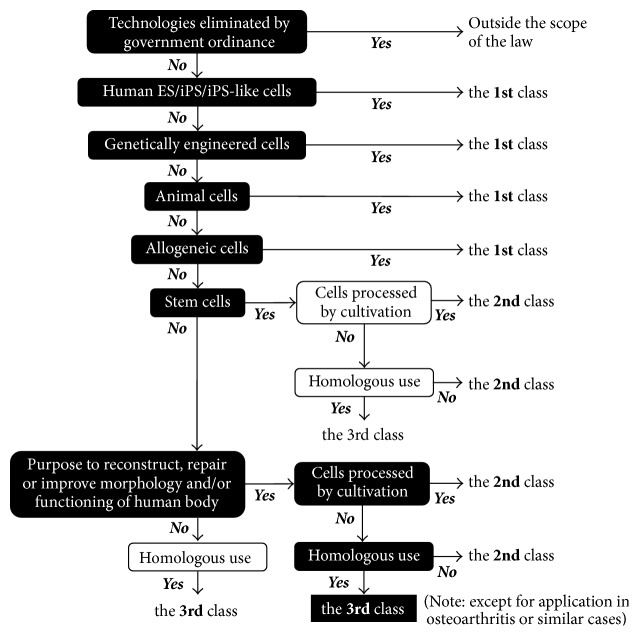
Classification of PC therapies in accordance with the risk tree presented by the Ministry of Health, Labour and Welfare of Japan.

For maintenance and improvement of health of aged individuals, a new regulatory framework has been applied to regenerative medicine, and, for this purpose, two laws known as the “Act on the Safety of Regenerative Medicine” and the “Pharmaceuticals, Medical Devices and Other Therapeutic Products Act” came into effect on November 25, 2014 [7–9]. On the basis of the former law, the Ministry of Health, Labour and Welfare of Japan (MHLW) established a regulatory framework necessary for examination of the regenerative-medicine provision plan and for inspection of cell culturing and processing facilities, aiming to ensure full safety of regenerative medicine and related modalities and to promote the development of its practical applications [8]. In these regulations, MHLW classifies cell-based therapy into three categories (Table 1); however, medical technologies specified by the government ordinance, that is, blood transfusion, hematopoietic stem cell transplantation, and assisted reproduction, are excluded from these regulations. Their principles and essence have been described and discussed in detail in several articles [7, 10, 11]; however, no convincing legal basis for classification of PCs has been provided. According to the risk tree presented by MHLW, we can recognize that regenerative and aesthetic therapies using PCs are categorized into “the 3rd class” under the regulations (Figure 1). Please check a series of criteria written in white inside black boxes to follow the classification process of PC therapy. To our knowledge, these regulations are the strictest for the use of PCs in regenerative and aesthetic therapies among medically advanced countries. Although it is hard to predict how many regulatory authorities in individual countries will follow Japan's actions, it would be beneficial for patients to improve the clinicians' knowledge and awareness of the safety of “home-made biologicals” and ways to minimize possible adverse effects and complications.

Here, the definition of “processing” should be mentioned: this term is defined as procedures related to artificial cell growth, differentiation, immortalization, and activation, among other methods. Nonetheless, the simplest procedures, such as separation and mincing of tissues and/or cells (note: chemical-reagent–dependent separation techniques are excluded), treatment with antibiotics, *γ*-ray irradiation, freezing, and thawing, shall not be deemed “processing” as defined by these regulations. Nevertheless, when cells are processed to have a structure and function that are different from their original ones regardless of the criteria described above, this procedure shall be deemed “processing” as defined by these regulations. Preparation of PCs always includes centrifugation and sometimes anticoagulants and coagulation factors. Although this procedure of course is not aimed at forcing platelets and leukocytes to grow, differentiate, or get immortalized, platelets can be more or less activated by centrifugation and coagulation factors. In public interpretation, the crucial criterion for PCs is probably “the purpose of processing,” that is, the purpose of inducing expression of supraphysiological structure and function of platelets.

It was not and is not easy for many clinicians to accept and learn this new regulatory framework because they have never been educated or trained to understand the basic concepts of CBMPs, good manufacturing practice (GMP), and/or standard operating procedure- (SOP-) based preparation and treatment. On the other hand, we have to accept that the world is changing. In general, clinicians' discretion tends to be more restricted now than before, and their decisions now tend to be controlled in detail by specific regulatory arrangements, especially in the field of advanced medical care, including cell-based therapy.

## 3. Quality of PCs

### 3.1. Quality of CBMPs: A General Concept

In general, CBMPs should be prepared at licensed facilities in accordance with the licensed SOPs based on the concepts of GMP. Haddock et al. [13] mentioned that determining a specific strategy for the creation of a GMP facility for cell manufacturing will depend largely on the appropriate business model and the type of cell product. Whether a bedside (i.e., chairside in dentistry) point-of-care manufacturing model (e.g., for autologous cells), a regional manufacturing hub model, or a centralized manufacturing model is the most appropriate must be decided a priori based on patients' needs, transportation and storage options, cost, and flexibility of the product. In addition to clinical manufacturing facilities, there is a great need for GMP (or at least, GMP-like) facilities for product and process verification and validation as well as workflow simulation. In the current situation, major PC preparations are regarded as products generated by means of a point-of-care manufacturing model without transportation or storage. By contrast, with changes in patients' and/or clinicians' needs in the near future, this manufacturing style may change to the regional manufacturing hub model or something similar. In any case, each patient's CBMPs should be considered one batch, and quality of each batch should be ensured before shipping; this approach is necessary to meet current GMP (cGMP) requirements and to get PCs integrated into safe and effective cell-based therapeutics [14].

### 3.2. What Is Included in the “Quality” of CBMPs

**Figure 2:**
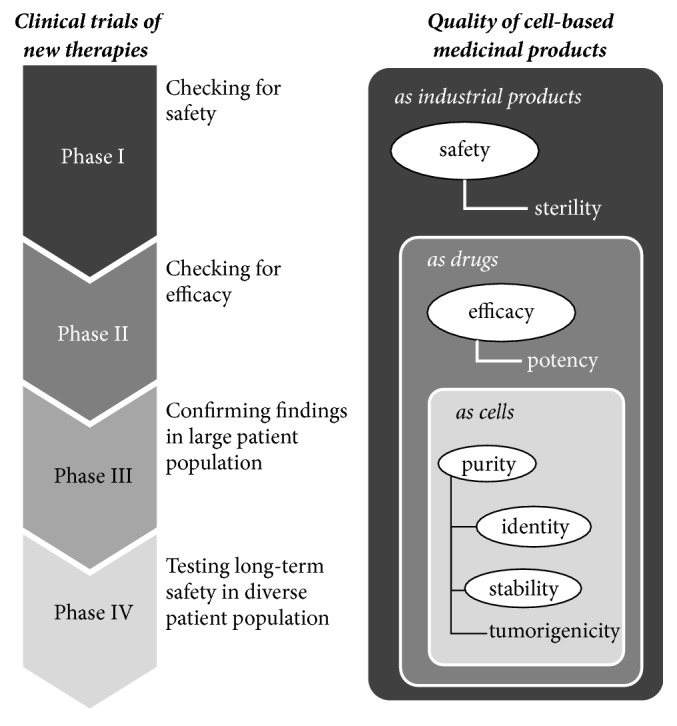
Illustration of general concept for quality of CBMPs and a flowchart of clinical trials for new therapies as reference.

Quality is subdivided roughly into two closely related issues: safety and efficacy, which are further classified into sterility, purity, identity, potency, and stability [15, 16]. We illustrated this concept with the flowchart of clinical trial in Figure 2. In clinical trial, safety and efficacy are examined successively at Phases I and II, respectively. Similarly, in quality assurance, safety is the main criterion for industrial products in the “broadest and the highest” concept, and efficacy is for conventional types of drugs in the “moderate” concept under the broadest concept. Purity, identity, and stability are also related to drug quality; however, in CBMPs, these criteria are closely related to tumorigenicity. Stem cells, which include pluripotent to multipotent stem cells processed for cell-based therapy, should essentially and ideally be examined prior to every shipping to a clinical organization or entity. When preoperative examinations cannot be performed in a timely manner, for example, in case of cell-specific problems, for example, chromosomal abnormality and tumorigenicity, only randomly selected cell product batches have been tested or the corresponding cell populations in parallel cultures have been evaluated after shipping. Nevertheless, this standardized examination procedure is intended for quality assurance of multipotent nucleated cells and may also be applied to other somatic cells. It is questionable from several biological standpoints (described in the next section) whether PCs should be regulated by the same criteria. According to the major differences between somatic stem cells and platelets or leukocytes in PC preparations (Table 2), platelets and leukocytes are clearly distinguishable from stem cells in the capacity for proliferation and differentiation.

### 3.3. General Quality of Prepared PCs

It has generally been accepted that quality—in a broad sense—of PC preparations is more or less influenced by devices employed and an operators' technique. The factors influencing PC quality are summarized in Table 3. When a needle is made of metal, of a narrow gauge, and/or long, platelets are likely to be easily activated to form aggregates in the absence of anticoagulants. A centrifugation protocol is well known to influence the quality of PC preparations in the absence of anticoagulants [17]. In particular, because contact of blood with the inner glass wall of a blood-collection tube triggers coagulation [18], the material of the tube and duration, force, and direction (angle) of contact severely affect the quality of platelet-rich fibrin (PRF) preparations. Furthermore, even though a blood-collection tube is made of glass, it should be noted that the inner surface is usually coated with silicone or a similar polymer to improve biocompatibility. However, thickness and quality of this surface coating vary among individual products and individual lots of the same product depending on the proficiency of a manufacturer. In fact, we and other clinician groups have seen incomplete coagulation in imported off-brand glass tubes that are not licensed as a medical device in Japan. Additionally, blood-collection tubes are generally produced for laboratory experiments, not for preparation of implantable blood-derived materials. Therefore, clinicians should primarily employ tubes approved for preparation of PCs by authorized agencies of individual countries. If such products are not commercially available, clinicians should choose products manufactured in accordance with reliable quality systems of manufacturing, that is, by GMP facilities. For all countries around the world, especially for European countries, the CE mark may be a reliable standard of quality. Therefore, CE-labeled tubes are expected to function in the same way in the same products with respect to processing specifications or standards, thereby simplifying and favoring the use of PCs [4].

In addition, as for preparation of PRP from citrated whole blood, an operator's technique and choice of coagulation factors are highly important and influence quality. The use of ethylenediamine tetra-acetic acid (EDTA) in preparations of PRP is potentially more harmful, and large numbers of damaged platelets have been observed [19]. A trisodium citrate solution is an anticoagulant with no negative effects on PRP preparations and, consequently, acid-citrate-dextrose (ACD) is a preferred anticoagulant.

Poor pipetting skills may induce undesirable platelet activation and reduce platelet concentration, whereas added thrombin produces a fibrin matrix composed of relatively thin fibrin fibers [20].

### 3.4. Safety, Stability, Purity, Sterility, Identity, and Potency of PCs

In this subsection, we discuss these evaluation indicators for autologous PCs because allogeneic PCs are not generally accepted for regenerative therapy at present. In general, PCs are prepared from autologous whole-blood samples essentially in accordance with the licensed SOPs. Nevertheless, basically and theoretically, the safety of autologous PCs is supported by the following factors. First, autologous PCs are immunologically neutral and pose no danger of allergy, hypersensitivity, or foreign-body reactions. Second, because of autologous implantation, there are no worries about the transmission of infectious diseases, neoplastic cells, or other unknown factors. Third, regardless of origin, their major cell components are platelets and leukocytes: platelets are anucleate cells, whereas leukocytes, despite being nucleated cells, are highly differentiated cells and manifest no spontaneous growth activity (i.e., show high stability). Fourth, regardless of origin, PCs are subjected to only gravity-based fractionation and activation, which are considered minimal manipulations. Taken together with our clinical experience in the last 25 years in regenerative dentistry, the above observations suggest that the probability of either tumorigenicity, that is, strong instability, or other possible adverse effects can be ignored.

Despite being heterogeneous, that is, not purified, blood cell populations, as long as PCs are prepared aseptically, their sterility and identity can be ensured. Their potency can be evaluated by several in vitro assays that do not require much time and is roughly proportional to the platelet count [21]. Therefore, point-of-care determination of platelet counts in prepared PCs is recommended as a minimum check point for efficacy assurance. If hematological analyzers are not available on site, just as bacterial counts, platelet counts in PRP can be conveniently and inexpensively determined by the spectrophotometric method at 615 nm (Kitamura et al., manuscript in submission). Furthermore, it is noteworthy that platelet counts in nonliquid products, such as PRF preparations, can also be determined accurately by our recently developed digestion method [22].

### 3.5. Systemic Conditions Influencing Quality of Whole-Blood Samples and Resulting PCs

PC therapy is widely employed as an adjuvant modality in different surgical procedures, more frequently in the areas of dentistry and orthopedics [1, 2, 23]. Therefore, variations in clinical cases and surgeon's techniques, in addition to those among individual samples, are considered more influential than variations in devices and tubes. Thus, as reported elsewhere [17, 24, 25], randomized controlled trials (RCTs) assessing limited numbers of clinical cases usually have low quality and cannot reach conclusive results on outcomes of PC therapy.

We propose that the general condition of a patient should be more carefully and precisely evaluated via his/her medical history and selected preoperative systemic analyses, especially blood tests. Blood coagulation is regulated mainly by the interactions of coagulation factors and platelets. Therefore, even though platelet counts may be substantially lower than the normal range, or platelet activity can be significantly suppressed, sufficient coagulation activity may compensate for these drawbacks. In this case, however, it is plausible that growth factors may not be concentrated at expected levels. In contrast, when coagulation activity is substantially lower than normal, compensation by platelets may be less adequate than expected.

These changes can often be induced by a disease, medication, mental or physiological stress, or other factors. Marques et al. [23] stated that there are some conditions where the indication must be followed with caution, as in cases of thrombocytopenia, platelet dysfunction syndrome, septicemia, hypofibrinogenemia, a recent febrile condition, anemia, cancer, skin lesions in the area of the injection, use of corticosteroids (up to 2 weeks before the procedure), nonsteroidal anti-inflammatory drugs (up to 48 hours before the procedure), or an active infection with* Pseudomonas*,* Klebsiella*, or* Enterococcus*.

To give examples that are more familiar to many clinicians, warfarin and aspirin reduce coagulation and platelet activities, respectively [26–29]. Type 2 diabetes mellitus is characterized by hemostasis dysfunction caused by endothelial anomalies and platelet hyperreactivity [30, 31]. Diet and nutrients have been demonstrated to influence platelet function [32, 33] although these alterations may usually be below severe pathological levels. In addition, mental or physical stress activates platelets to induce thrombosis [34–38]. Possible effects of these systemic aberrations on quality of prepared PCs have not been clarified and need to be investigated to more precisely evaluate suitability of platelets and coagulation systems and to establish the cut-off values of several parameters to be inspected, such as a platelet count, platelet aggregation activity, prothrombin time, thrombin time, and coagulation time, for preparation of quality-assured PCs.

## 4. General Risk Factors in Cell-Based Therapy and Possible Risks of PC Therapy

As described in the earlier section, autologous PCs are immunologically neutral and carry no risk of transmission of microbial pathogens. Nonetheless, the efficacy of individual PC preparations is not always at or above standard levels that are sufficient for inducing tissue regeneration. In this section, we again analyze quality of PC therapy in terms of additional clinical criteria, risk factors, and risks. In comparison with stem cell therapy [12] and on the basis of risk factors associated with stem cell therapy, possible risks of PC therapy are listed in Table 4. The risk factors are categorized into three groups: intrinsic, extrinsic, and clinical factors. In any category, possible risks of PC therapy boil down to sterility and efficacy issues, which are considered minor risks compared to those of stem cell therapy. Therefore, also from the viewpoint of risk factors and risks, PC therapy can be regarded as the safest cell-based therapy.

## 5. Possible Case-Specific Risks Related to Contraindications

Neither severe complications nor adverse effects of PC therapy have been reported in the past 25 years in the frontier field of regenerative medicine: regenerative dentistry. Consequently, there is a consensus that possible tumorigenicity or a severe complication can be ruled out; however, we are wondering whether those accumulated clinical data are sufficient to completely ignore any unknown hidden risks of PC therapy and to guarantee its safety for topical use. Here, we will voice several concerns. First, several in vitro studies have revealed that mitogenic action of leukocyte-rich PRP is not necessarily proportional to platelet counts and that an optimal concentration is approximately 2.5% in the culture medium or 2.5-fold above the basal levels [39, 40]. This result implies that an unidentified adverse factor(s), probably as well as inflammatory cytokines released by leukocytes, may act on cells nonspecifically and toxically in case of overdosing. Second, when a PC is combined with other biomaterials, for example, animal-derived, less characterized, but frequently applied bone substitutes, who can guarantee the safety of PC therapy? Is it possible to rule out the risk that PCs augment an unfavorable action of unidentified factors resulting from these biomaterials? This is off-label use of PCs. Third, why are the efficacy and clinical outcomes largely dependent on a host's topical and systemic conditions? That is the reason why PCs are considered adjuvant. Further basic studies are needed to clarify possible case-specific risks and disadvantages of PC therapy.

## 6. Indications and Contraindications

To administer a predictable or at least adequate PC therapy, it is important to have criteria for appropriate selection of patients and cases. In regenerative dentistry, relatively large alveolar bone defects may not be considered an appropriate indication for therapy by means of PC preparations alone. According to the currently accepted therapeutic strategy, it is better for clinicians to combine PCs with well-defined scaffolding materials and/or osteogenic cells for large bone defects [41, 42]. Even in case of small bone defects, if patients have a tumor in surrounding regions, it may be better to choose alternative therapeutic strategies rather than PC therapy. PC administration, as well as surgical stress, is thought to cause tumor tissues to grow faster because PCs contain various angiogenic factors [43]. In contrast, because of poor anatomical and physiological characteristics, tissue regions with weaker angiogenic properties, for example, a peri-implant region of gingival tissue and the relatively wide and flat region of the maxillary sinus, may not be suitable for successful regeneration by PC therapy [44].

As for systemic conditions, Sampson et al. mentioned in their article [45] that moderate contraindications of PC therapy include the presence of a tumor, metastatic disease, an active infection, or a platelet count < 10^5^/*μ*L with hemoglobin < 10 g/dL. Pregnancy or active breastfeeding is contraindication. Patients with an allergy to bupivacaine (Marcaine) should not receive local anesthesia involving these substances.

To our knowledge, these kinds of data have not been well systematized in a useful data bank like the Japanese Adverse Drug Event Report database of the Pharmaceutical and Medical Devices Agency that are available to the public. To build this kind of a database, individual governments, relevant international medical societies, and/or public organizations should function as a common platform to begin collection and analysis of clinical data, then propose both indications and contraindications, and finally should test the validity of this classification prior to dissemination of the above information to the public. In advance of or in parallel with building the database, we should further develop sensitive but convenient methods for evaluation of topical and systemic conditions.

Several clinicians and researchers, including our group, have repeatedly proposed the necessity of standardized preparation protocols and therapeutic procedures, for example, in terms of timing and dosing [17, 23–25, 43]. However, we recently came to realize that the priority of research should be shifted from standardization of PC preparation and applications to clarification of indications and contraindications for successful PC therapy.

## 7. Conclusion

To date, high-quality RCTs with large numbers of human subjects have not been conducted to evaluate PC therapy in terms of both safety and efficacy regardless of specific fields of regenerative medicine. Contradictory results regarding efficacy have always been attributed to the differences in individual samples and preparation protocols without careful consideration or discussion, while safety has been endorsed seemingly without careful or long-term standardized evaluation. Both clinicians and basic researchers have paid much attention to PC efficacy and have proposed standard preparation protocols (to produce similar PC preparations) and universal terminology of PCs to more precisely compare their clinical outcomes [17]. In previous articles [43, 46], we independently proposed the necessity of standardized protocols for PRF preparation and of terminology based on generic rather than vendor names. In this review article, we continue to emphasize the need for rerecognition of “safety myths” of PC therapy and propose performing a specific blood test in the spirit of GMP-based manufacturing and surveying patients' medical history more carefully than usual prior to PC therapy. We believe that standardization of such a series of preoperative assessments is urgently needed and that this preoperative examination will reduce inappropriate therapeutic use of PCs and simultaneously improve general quality of this modality.

## Figures and Tables

**Table 1:** Classification of cell-based therapies by their potential risks.

Class	Definition	Example
1st	High-risk therapy involving cells with little or no supporting clinical evidence	ES cells, iPS cells
2nd	Medium-risk therapy involving cells currently being implemented in clinical settings	Somatic stem cells
3rd	Low-risk therapy involving cells that are subjected to limited manipulation or processing	Somatic cells

This table was translated from the original presented in the ministry's guidance document [9]. ES cells: embryonic stem cells. iPS cells: induced pluripotent stem cells.

**Table 2:** Characteristics of somatic stem cells, platelets, and leukocytes.

	Somatic stem cells	Platelets	Leukocytes
Isolation	Postnatal adult tissue	Adult venous blood	Adult venous blood
Origin	Autologous or allogeneic material	Autologous	Autologous
Differentiation potential	Multipotent	No	No
Differentiation lineage	Limited number of cell types depending on tissue of origin	No	No
Chimera formation	No	No	No
Self-renewal	Limited	No	No
Maintenance in cell culture	Difficult for long periods	No	Possible
Ease of access, yield, and purification	Depending on source tissue	Easy	Easy
Lifespan	Limited	Limited	Limited
Production efficiency	Limited number of cells (chromosome tend to shorten with ageing)	Efficient Fractionation	Efficient Fractionation
Risks of treatment	No	No	No
Ethical issue	No	No	No
Immunogenicity and immunomodulatory effects	Low immunogenicity and medium-to-strong immunomodulatory effects	No	No
Immune rejection	In case of autologous use, lower chance of immune rejection, but immunogenicity in allogeneic and nonhomologous applications remains unpredictable	No	No
Targeted disease	Targeted disease may still be present in stem cells in case of autologous use	No	No

Characteristics of somatic stem cells are taken from the literature [12] and adapted to PCs. It should be noted that, during preparation of PCs, undetectable levels or limited numbers of circulating hematopoietic stem cells may contaminate the collected blood samples, but these cells cannot be expanded during preparation or can hardly influence regeneration at an implantation site.

**Table 3:** Influential factors associated with quality of PCs.

Preparation step	Influential factor	Details
Blood collection	Needle	Gauge, length, material, surface modification
	Tubing of butterfly needle	Diameter, length, material
	Syringe/tube	Material, surface modification
	Lag	Distance between blood-collection space and centrifuge

Centrifugation	Tube	Shape, material, surface modification
	Rotator	Swing or angle
	Centrifugal condition	Force, duration

Other handling	Pipetting	Technique, material
	Coagulation	CaCl_2_, thrombin, glassware

**Table 4:** An overview of risk factors and risks associated with stem cell-based and PC-based therapy.

	Risk factors or hazards	Identified risks	
		Stem cell therapy	PC therapy
Intrinsic factors: cell characteristics	(i) Origin of cells (e.g., autologous versus allogeneic, diseased versus healthy donor or tissue) (ii) differentiation status (iii) tumourigenic potential (iv) proliferation capacity (v) lifespan (vi) long-term viability (vii) secretion patterns (e.g., growth factors, cytokines, chemokines) (viii) quality of cells (ix) quantity of cells	(i) Rejection of cells (ii) Disease susceptibility (iii) unwanted biological effect (e.g., in vivo differentiation into unwanted cell types) (iv) Toxicity (v) Tumor formation (benign or malignant)	(i) Lower efficacy (e.g., obtained from donor on medication or donor who has activated platelets, thrombocytopenia, or coagulation disorders) (ii) Exacerbation or metastasis of tumor

Extrinsic factors: manufacturing and handling	(i) lack of donor history (ii) starting and raw material (iii) plasma derived materials (iv) contamination by adventitious agents (viral/bacterial/mycoplasma/fungal species, prions, parasites) (v) cell handling procedures (e.g., procurement) (vi) culture duration (vii) tumorigenic potential (e.g., culture induced transformation, incomplete removal of undifferentiated cells) (viii) noncellular components (ix) pooling of allogeneic cell populations (x) conservation (e.g., cryopreservatives) (xi) storage conditions (e.g., failure of traceability, of human material labeling) (xii) transport conditions	(i) Disease transmission (ii) Reactivation of latent viruses (iii) Cell line contamination (e.g., with unwanted cells, growth media components, chemicals) (iv) Mix-up of autologous patent material (v) Tumor formation (benign or malignant)	(i) Bacterial contamination due to poor operator skills (ii) Irreversible inactivation of platelets at low temperature

Clinical characteristics	(i) therapeutic use (i.e., homologous or nonhomologous) (ii) indication (iii) administration route (iv) initiation of immune responses (v) use of immunosuppressants (vi) exposure duration (vii) underlying disease (viii) irreversibility of treatment	(i) Undesired immune response (e.g., GVHD) (ii) Unintended physiological and anatomical consequences (e.g., arrhythmia) (iii) Engraftment at unwanted location (iv) Toxicity (v) Lack of efficacy (vi) Neoplasm formation (benign or malignant)	(i) Lower efficacy (e.g., in diabetic patient)

Risk factors and identified risks of stem cell therapy are taken from the literature [12] and adapted to PC therapy. GVHD: graft-versus-host disease.

## Abbreviations

PCs: Platelet concentrates
PRP: Platelet-rich plasma
PRF: Platelet-rich fibrin
MHLW: The Ministry of Health, Labour and Welfare of Japan
CBMP: Cell-based medicinal product
GMP: Good manufacturing practice
EDTA: Ethylenediamine tetra-acetic acid
ACD: Acid-citrate-dextrose
RCT: Randomized controlled trial
SOP: Standard operating procedure
ES cells: Embryonic stem cells
iPS cells: Induced pluripotent stem cells
GVHD: Graft-versus-host disease.
